# Supplementary material for: A Novel Mineral-like Copper Phosphate Chloride with a Disordered Guest Structure: Crystal Chemistry and Magnetic Properties
Source: Materials (Basel). 2022 Feb 14;15(4):1411. doi: 10.3390/ma15041411 (PMC8876330; doi:10.3390/ma15041411)
Supplement: Supplementary file 1 [file materials-15-01411-s001.zip › materials-1538266-supplementary.pdf]

materials

Supplementary Information

A Novel Mineral-like Copper Phosphate Chloride with a Disordered Guest Structure: Crystal Chemistry and Magnetic Properties

Galina Kiriukhina [1,2], Olga Yakubovich [1], Larisa Shvanskaya [1,3], Anatoly Volkov [1], Olga Dimitrova [1], Sergey Simonov [4], Olga Volkova [3,5,6] and Alexander Vasiliev [3,5,6]*

1 Faculty of Geology, Lomonosov Moscow State University, 119991 Moscow, Russia; g-biralo@yandex.ru (G.K.); yakubovich.olga320@gmail.com (O.Y.); lshvanskaya@mail.ru (L.S.); toljha@yandex.ru (A.V.); dimitrova@list.ru (O.D.)
2 Institute of Experimental Mineralogy RAS, 142432 Chernogolovka, Russia
3 Quantum Functional Materials Laboratory, National University of Science and Technology "MISiS", 119049 Moscow, Russia; os.volkova@yahoo.com
4 Institute of Solid State Physics RAS, 142432 Chernogolovka, Russia; simonov@issp.ac.ru
5 Faculty of Physics, Lomonosov Moscow State University, 119991 Moscow, Russia
6 Institute of Physics and Technology, Ural Federal University, 620002 Ekaterinburg, Russia
* Correspondence: anvas2000@yahoo.com

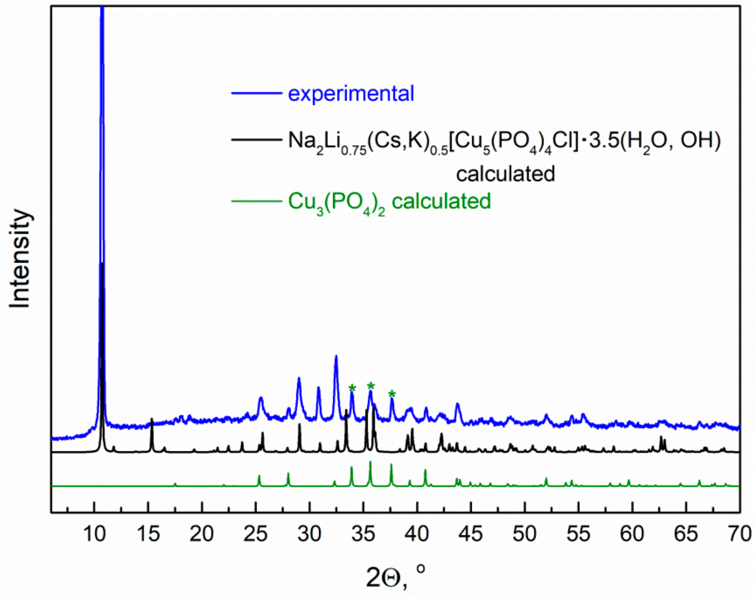

Figure S1. Experimental and calculated powder XRD patterns. Peak marked with asterisk corresponds to the impurity phase, $Cu_3(PO_4)_2$. The powder XRD analysis was performed by an ADP-2 diffractometer using CoKα radiation, λ=1.7903 Å.

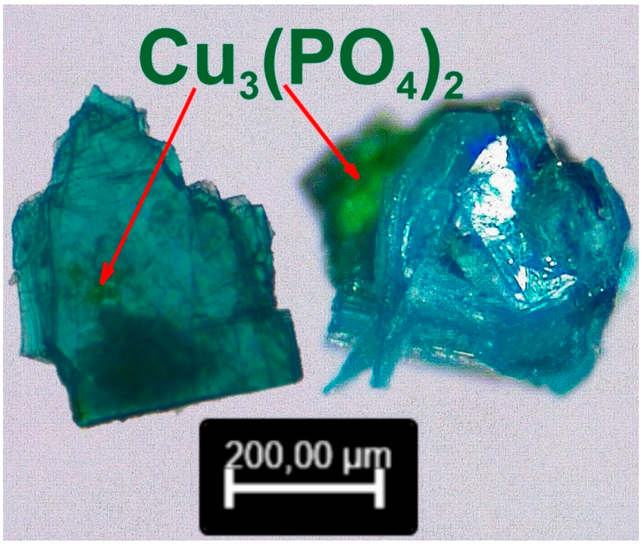

Figure S2. Intergrowth of $Na_2Li_{0.75}(Cs,K)_{0.5}[Cu_5(PO_4)_4Cl]\cdot 3.5(H_2O,OH)$ (blue) and $Cu_3(PO_4)_2$ phase (green).

Table S1. Fractional atomic coordinates and isotropic or equivalent isotropic displacement parameters (A^2) for $Na_2Li_{0.75}(Cs,K)_{0.5}[Cu_5(PO_4)_4Cl]\cdot 3.5(H_2O,OH)$.

	x	*y*	*z*	U_{iso}*/U_{eq}	Occ.
Cu1	0.59537(6)	0.5000	0.20041 (11)	0.0041 (2)	1
Cu2	0.71493(6)	1.0000	0.50829 (11)	0.0048 (2)	1
Cu3	0.71529(6)	1.0000	0.96873 (11)	0.0054 (2)	1
Cu4	0.71506(4)	0.77095 (8)	0.73848 (8)	0.00537 (18)	1
Cl1	0.63652(12)	1.0000	0.7144 (2)	0.0096 (4)	1
P1	0.66048(9)	0.70784 (16)	0.42766 (16)	0.0048 (3)	1
P2	0.66045(9)	0.70858 (16)	1.01322 (16)	0.0049 (3)	1
Cs1	0.5000	0.8213 (5)	0.5000	0.0473 (13)	0.334 (6)
K1	0.5000	0.894 (3)	0.5000	0.0473 (18)	0.195 (11)
O12	0.5000	1.0000	0.5000	0.07 (3)*	0.24 (6)
Na2	0.6077 (4)	1.0000	0.2053 (8)	0.0104 (15)	0.980 (3)
Cu6	0.711 (3)	1.0000	0.289 (6)	0.0104 (15)	0.020 (3)
Na1	0.6110 (2)	0.5000	0.6953 (4)	0.0098 (8)	0.979 (3)
Cu5	0.404 (3)	0.5000	0.471 (6)	0.0098 (8)	0.021 (3)
O1	0.7276 (2)	0.6423 (5)	0.3866 (4)	0.0052 (9)	1
O2	0.7272 (2)	0.6426 (5)	1.0987 (4)	0.0058 (9)	1
O3	0.5954 (2)	0.6434 (5)	1.0567 (4)	0.0078 (9)	1
O4	0.5962 (2)	0.6428 (5)	0.3426 (5)	0.0074 (9)	1
O5	0.6606 (2)	0.8620 (5)	0.3956 (4)	0.0071 (6)	1
O6	0.6602 (2)	0.6767 (5)	0.8591 (4)	0.0077 (9)	1
O7	0.6605 (2)	0.8628 (5)	1.0452 (5)	0.0071 (9)	1
O8	0.6602 (2)	0.6777 (5)	0.5824 (4)	0.0071 (6)	1
O9	0.4775 (4)	0.5000	0.1629 (7)	0.0104 (14)	1
O11	0.5000	0.5000	0.5000	0.021 (2)	1
O10	0.5071 (4)	0.8408 (8)	0.1824 (12)	0.071 (3)	1
Li1	0.5000	0.806 (3)	1.0000	0.026 (10)	0.74 (8)
Cl2	0.591 (4)	1.0000	0.189 (9)	0.013 (17)*	0.069 (13)
H1	0.466 (4)	0.433 (3)	0.114 (4)	0.02 (2)*	1

Table S2. Interatomic distances for $Na_2Li_{0.75}(Cs,K)_{0.5}[Cu_5(PO_4)_4Cl]\cdot 3.5(H_2O,OH)$.

Cu1 pyramid		Cu2 pyramid		Cu3 pyramid	
Cu1 O4	1.963(4) × 2	Cu2 O5	1.938(5) × 2	Cu3 O7	1.933(4) × 2
Cu1 O3	1.979(4) × 2	Cu2 O1	1.962(4) × 2	Cu3 O2	1.962(4) × 2
Cu1 O9	2.255(7)	Cu2 Cl1	2.708(2)	Cu3 Cl1	2.691(3)
**Cu4 pyramid**		**P1 tetrahedron**		**P2 tetrahedron**	
Cu4 O8	1.936(4)				
Cu4 O6	1.940(4)	P1 O4	1.519(5)	P2 O6	1.532(4)
Cu4 O1	1.969(4)	P1 O8	1.537(4)	P2 O3	1.533(5)
Cu4 O2	1.975(4)	P1 O5	1.537(5)	P2 O7	1.537(5)
Cu4 Cl1	2.6944(15)	P1 O1	1.559(4)	P2 O2	1.561(5)
**Cs1 polyhedron**		**K1 polyhedron**		**Na1 polyhedron**	
Cs1 O4	3.130(5) × 2				
Cs1 O11	3.137(5)	K1 O10	3.162(13) × 2	Na1 O8	2.338(5) × 2
Cs1 O10	3.124(11) × 2	K1 Cl1	3.262(11) × 2	Na1 O9	2.372(8)
Cs1 O8	3.385(5) × 2	K1 O5	3.44(2) × 2	Na1 O6	2.436(5) × 2
Cs1 O5	3.452(4) × 2	K1 O4	3.57(2) × 2	Na1 O11	2.632(4)
Cs1 Cl1	3.553(3) × 2				
**Na2 trigonal prism**		**Cu5 polyhedron**		**Cu6 polyhedron**	
Na2 O5	2.385(8) × 2	Cu5 O11	1.84(6)	Cu6 O5	2.04(4) × 2
Na2 O7	2.407(8) × 2	Cu5 O8	2.15(3) × 2	Cu6 O7	2.76(4) × 2
Na2 O10	2.475(10) ×2	Cu5 O4	2.29(4)	Cu6 Cl2	2.36(9)
Li tetrahedron	Li O10	1.760(14) ×2			
	Li O3	2.44(2) × 2			

Table S3. Hydrogen-bond geometry (Å, º) for $Na_2Li_{0.75}(Cs,K)_{0.5}[Cu_5(PO_4)_4Cl]\cdot 3.5(H_2O,OH)$.

D—H···*A*	*D*—H	H···*A*	*D*···*A*	*D*—H···*A*
O9—H1···O3^{i}	0.82 (1)	2.02 (3)	2.748 (7)	148 (6)

Symmetry code: (i) $-x+1$, $-y+1$, $-z+1$
